# Instant Dark Tea Alleviates Hyperlipidaemia in High-Fat Diet-Fed Rat: From Molecular Evidence to Redox Balance and Beyond

**DOI:** 10.3389/fnut.2022.819980

**Published:** 2022-02-03

**Authors:** Si Qin, Zhilan He, Yuanjie Wu, Chaoxi Zeng, Zhibing Zheng, Haowei Zhang, Chenghao Lv, Yong Yuan, Haoren Wu, Jianhui Ye, Zhonghua Liu, Meng Shi

**Affiliations:** ^1^Lab of Food Function and Nutrigenomics, College of Food Science and Technology, Hunan Agricultural University, Changsha, China; ^2^Hunan Tea Group Co. LTD, Changsha, China; ^3^Tea Research Institute, Zhejiang University, Hangzhou, China; ^4^Key Laboratory of Ministry of Education for Tea Science, Hunan Agricultural University, Changsha, China

**Keywords:** dark tea, composition, oxidative homeostasis, lipid metabolism, gut microbiota, *Akkermansia*

## Abstract

Instant dark tea (IDT) is a new product gaining increasing attention because it is convenient and can endow significant health benefit to consumers, which is partially attributed to its high concentration of functional ingredients. However, the molecular mechanism underlying its regulatory effect on hyperlipidaemia is rarely studied. In this study, we performed omics and molecular verification in high-fat diet (HFD)-fed rat, aiming to reveal the mechanism and provide molecular evidence. The results showed that the major bioactive components in IDT were 237.9 mg/g total polysaccharides, 336.6 mg/g total polyphenols, and 46.9 mg/g EGCG. Rats fed with IDT (0.27–0.54 g/kg for 12 weeks) significantly reduced the body weight and TC, TG, LDL-C, blood glucose, and MDA and induced the level of serum HDL-C and also the levels of liver SOD, CAT, GSH-Px, and Nrf2, compared to HFD group. For molecular mechanism study, HIDT feeding had significant impact on the gene expressions of biomarkers in lipogenesis (*FABP, CD36, SCD1, Cyp4a1*, and *Kcnn2*), lipid oxidation (*PPAR*γ), and glucose glycolysis (*Gck* and *ENO2*) in liver tissue. Moreover, gut microbiome study found that rats fed with IDT dramatically modified the gut microbial species at the family level, such as suppressing the increase abundance of Proteobacteria and Firmicutes induced by HFD. HIDT significantly boosted the relative composition of beneficial bacterium *Akkermansia* and *Rikenellaceae_RC9_gut_group* and decreased the relative abundance of the harmful bacterium *Ruminococcaceae_UCG-005* and *Ruminiclostridium_9*, compared to HFD (*p* < 0.01). Correlation analysis between microbiome and animal indicators found that seven genera including *Akkermansia, Clostridiales, Lachnospiraceae, Lachnospiraceae_UCG-010, Ruminiclostridium_9, Ruminococaceae-UCG-005*, and *Ruminocuccus_1* were found as potential biomarkers that were strongly correlated with oxidative stress and metabolism genes. For instance, *Ruminococcaceae_UCG-005* was significantly correlated with body weight, TG, HDL-C, Nfr2, *FABP3, SCD1, Cyp4a1*, and *Kcnn2*. Collectively, the above data obtained in this study had provided the primary molecular evidence for the molecular mechanism and brought in novel insights based on omics for the regulatory effect of IDT on hyperlipidaemia.

## Introduction

Hyperlipidaemia is a metabolic disorder characterized by elevated levels of total cholesterol (TC), triglyceride (TG), and low-density lipoprotein cholesterol (LDL-C) and a reduced concentration of high-density lipoprotein cholesterol (HDL-C) in the plasma (1). Abnormal lipid metabolism is the dominant causing of chronic diseases including obesity, diabetes, and cardiovascular and cerebrovascular diseases (2). The underlying mechanism of this action is strongly linked to the diet and alterations of gut microbiota (3). Diet with high-fat consumption causes imbalance of oxidative stress, change in lipid metabolism, and microbial dysbiosis, resulting hyperlipidaemia (4). ROS is the major oxidative factor, which could be dramatic increased in liver under high-fat diet (HFD) (5). The endogenous antioxidant molecules such as superoxide dismutase (SOD), catalase (CAT), and glutathione peroxidase (GSH-Px) play important roles in alleviating tissue damage caused by free radicals (6). However, the excessive formation of ROS induced by hyperlipidaemia can overcome the endogenous antioxidant defense system, which leads to oxidative stress reactions such as lipid peroxidation.

Hepatic lipid metabolism is crucial for the controlling of the lipid homeostasis in whole body. Lipid metabolism is a complex process involved in the regulation of some crucial nuclear factors, such as fatty acid-binding proteins (*FABPs*), peroxisome proliferator-activated receptor γ (*PPAR*γ), *CD36*, and *SCD1*(7). *FABPs* modulate lipid fluxes, trafficking, signaling, and metabolism (8). The deficiency of *FABP* increased *PPAR*γ activity in macrophages, which results in elevated expression of target genes. *PPAR*γ, a nuclear receptor that acts as a ligand-inducible transcription factor that regulates fat storage and glucose homeostasis, exerting antioxidant and antiinflammatory effects, is downregulated by diet-induced obesity (9, 10). *SCD1* is one of the downstream effectors of *PPAR*γ, which was reported significantly induced after HFD, accompanied by lipid accumulation (11). *CD36* not only acts as a free fatty acid (FFA) transporter, but also regulates FFA oxidation, lipid synthesis, VLDL secretion, inflammation, and autophagy in liver cells (12). Gut microbiota plays an important role in the host lipid metabolism and health. For example, Firmicutes or Bacteroidetes ratio was reported significantly enhanced in obesity in comparison with normal subjects (13). Thus, targeting on the hepatic lipid metabolic gene and gut microbiota was a key strategy for treating hyperlipidaemia. Although the hyperlipidaemic patients regularly take drugs, such as statins and fibrates, they may have significant adverse effects or contraindications (14). This leads to a critical public health goal for developing natural alternative in preventing hyperlipidaemia.

It is extensively accepted that dark tea has been consumed for hundreds of years in China, presenting plenty of beneficial effects especially for lipid-lowering, antiobesity, and cardiovascular protective (15, 16). The biological function of dark tea is attributed by the functional components such as polysaccharides and polyphenols, and also the newly formed functional components fermented by important microorganism such as *Eurotium cristatum*. Dark tea was found to possess the strongest *in vivo* antioxidant and hepatoprotective activities in mice among all six types of tea targeting on oxidative stress (17). Notably, fat accumulation was reduced and the genes related to FFA uptake and β-oxidation significantly changed after dark tea treatment (16). Besides, dark tea enhanced the growth of bacteria in obese mice associated with healthy metabolic markers including *Clostridiaceae, Bacteroidales, Lachnospiraceae, Akkermansia, Faecalibacterium prausnitzii, Lactobacilli, Actinobacteria, Muribaculaceae, Bacteroidaceae*, and *Prevotellaceae* and reduced in harmful bacteria comprising *Coriobacteriaceae, Streptococcaceae, Erysipelotrichaceae, Peptococcaceae, Peptostreptococcaceae*, and *Ruminococcaceae*, and also the ratio of Firmicutes to Bacteroidetes (F/B) (18–23).

Instant dark tea (IDT), a new soluble dry tea product made from dark tea, has been produced to meet the demand for modern society's convenience-oriented lifestyle, possessing lipid metabolism modulating effects (24). However, the essential role of IDT on gut microbiota has not been clarified, and there is a lack of investigation on the mechanism between lipid metabolism gene and gut microbiota. Therefore, this study examined the preventive effects of IDT against obesity and hyperlipidaemia in HFD-induced rats. Importantly, we attentively characterized the signaling pathways and gut microbiota involved in the prevention of lipid metabolism in rats fed with IDT. The results from this study may contribute to understand the role of IDT consumption in relieving obesity and lipid disorder-related syndromes of the interplay among gut microbiota in HFD-fed rat.

## Materials and Methods

### Materials and Chemicals

Instant dark tea was provided by Hunan Tea Industry Group Co. Ltd. (Changsha, China) with the fermentation of *Eurotium Cristatum*. Carboxylic acid (Trolox) and 2,2′-azinobis (3-ethylbenzothiazoline-6-sulfonicacid) diammonium salt (ABTS) were purchased from Shanghai Maclin Biochemical Reagent Co., Ltd. (Shanghai, China). Rutin, 2,2-diphenyl-1-picrylhydrazyl (DPPH), and phosphate buffer were purchased from Sinopharm Chemical Reagent Co. Ltd. (Shanghai, China). Fluorescein and 2,2′-azobis (2-methylpropionamidine) dihydrochloride (AAPH) were purchased from Aladdin Reagent Co. Ltd. (Shanghai, China). Folin-Ciocalteu reagent was purchased from Beijing Solarbio Science and Technology Co. Ltd. (Beijing, China). (-)-Epigallocatechin gallate (EGCG), (-)-epigallocatechin (EGC), (-)-gallocatechin gallate (GCG), (-)-epicatechin gallate (ECG), (-)-epicatechin (EC), (-)-gallocatechin (GC), (-)-catechin gallate (CG), (+)-catechin (C), theobromine (TB), theacrine, and gallic acid were purchased from Aladdin Industrial Co. Ltd. (Shanghai, China) with the HPLC purity ≥98%. Simvastatin was purchased from Tianjin Huairen Pharmaceutical Co. Ltd. (Tianjin, China).

### Composition and Antioxidant Activity of IDT

The total polyphenol content of IDT was determined by a modified Folin–Ciocalteu method with gallic acid as the standard (25). The content of total flavonoids in IDT was determined by colourimetric method of sodium nitrite-aluminum nitrate and strong sodium oxide using rutin as the standard (26). The content of amino acids was determined by amino acid assay kit following the manufacturer's instruction. The content of polysaccharides in IDT was determined by phenol–sulfuric acid method with anhydrous glucose as the standard (27), and the results were expressed as mg·g^−1^. DPPH, ABTS radical scavenging, and oxygen radical absorption capacity were assayed as described previously (28). Concentrations of catechins, gallic acid, caffeine, and theobromine were analyzed by the modified method described in the previous paper (29).

### Animals and Experiment Design

A total of 50 male Sprague Dawley (SD) rats with the body weight of 174.7–214.9 g were purchased from Hunan Slyke Jingda Laboratory Animal Ltd. (Changsha, China) and housed in the Hunan Research Center for Safety Evaluation of Drugs (SYXK2015-0016) in a controlled environment with room temperature at 22°C ± 2°C and a 12-h light–dark cycle. Rats were further randomly assigned to five groups (*n* = 10 per group), including control, HFD, simvastatin (SIM), low instant dark tea (LIDT), and high instant dark tea (HIDT). Control supplied with a basal chow diet (SCXK < Jing>2019-0003, Beijing Ke Ao Xie Li Feed Co. Ltd, China) and other groups supplied with a HFD (TP23300, containing 19.4% crude protein, 60.0% crude fat, and 20.6% carbohydrate, TROPHIC Animal Feed High-Tech Co. Ltd, China) for 12 weeks. The other three groups were fed HFD and administrated 0.002 g/kg body weight SIM, 0.27 g/kg body weight IDT (LIDT), and 0.54 g/kg body weight IDT (HIDT), respectively. The volume of oral gavage for each rat was 0.1 ml/10 g. All rats had free access to clean water and food. During the experimental period, rat's food intake and body weight were monitored and recorded once a week. After 8 weeks of feeding, rats were euthanised after 12 h fasting and quickly stored at−80 °C. The blood was collected by monocular enucleation. The blood samples were left at room temperature for 30 min and then centrifuged at 4,000 g for 10 min at 4 °C to collect serum. Livers were collected and trimmed off any adherent tissues. Serum samples and liver tissue were stored at −80 °C for further use.

### Histological Analysis and Morphometry

Part of the liver tissue was fixed with 4% paraformaldehyde. After 24 h, the livers were embedded in paraffin wax. Then, the liver samples were processed using cryostat (CM1950, Leica, Germany) and stained with haematoxylin–eosin (H&E). Finally, these slices were observed using the Olympus light microscope.

### Serum Parameters and Liver Index Analysis

The samples of serum were used to assess TC, TG, HDL-C, and LDL-C. Blood glucose test strips (Sinocare Inc Co. Ltd., Changsha, China) were used to measure blood glucose levels. Hepatic tissue (0.1 g) was homogenized with 1 mL ice-cold extract and centrifuged at 8,000 g for 10 min. The supernatant was collected for the analysis of hepatic ROS, MDA, CAT, and GSH-Px. All the detailed steps of these biochemical assessments were performed according to the instructions of corresponding commercial kits (Wako Pure Chemical Industries. Ltd., Japan; Beijing Solarbio Science &Technology Co. Ltd., Beijing, China; Hefei Laier Biotechnology Co. Ltd, Hefei, China).

### Liver Tissue Protein Extraction and Western Blot Analysis

Protein extraction from liver tissue was prepared according to the manufacturer's instruction of Total Protein Extraction Kit (Beijing Solarbio Science &Technology Co. Ltd, Beijing, China). Liver tissue was grinded with liquid nitrogen and lysed on ice. The supernatants containing protein extracts were stored at −80°C until use. Protein concentration was determined using BCA Protein Concentration Determination Kit (Beijing Solarbio Science &Technology Co. Ltd, Beijing, China). Protein extracts were separated by 10% SDS-PAGE and transferred to PVDF membranes (Amershan Pharmacia Biotech, Little Chalfont, UK). The membranes were blocked at room temperature for 1 h with 5% nonfat dry milk, then incubated with each primary antibody at 4°C overnight, and further incubated for 1 h with HRP-conjugated secondary antibody. Bound antibodies were detected by ECL system with a Lumi Vision PRO machine. Antibodies against Nrf2 were purchased from Abcam (Cambridge, MA, USA).

### RNA Preparation and QRT-PCR Analysis

To measure the expression of *FABP1, FABP3, FABP4, PPAR*γ, *CD36, SCD1, Cyp4a1, Kcnn2, Gck*, and *ENO2* in livers, total RNA was extracted by Omega Total RNA Kit (Omega Bio. Inc., Changsha, China). RNA was reverse-transcribed using PrimeScript RT Master Mix (TaKaRa Bio. Inc., Takara, Japan). The primers for qPCR were designed by Shengong Biological Engineering Co., Ltd. (Shanghai, China) according to cDNA sequences (Supplementary Table S1). Quantitative real-time reverse transcription PCR (qRT-PCR) was performed using SYBR Green Master Mix and QuantStudio 3 Flex (Thermo Fisher Scientific Inc., Shanghai, Beijing) according to the manufacturer's guidelines. Experiments were performed in triplicate in a single plate, and the relative quantification was calculated using the 2^−ΔΔCt^ method. Primer sequences for the targeted mouse genes were as follows: reactions were carried out with the Rotor-Gene Q6200 real-time PCR System (Qiagen) using three-step cycling conditions of 95°C for 10 min, followed by 40 cycles of 95°C for 10 s, 60°C for 20 s, and 72°C for 20 s. The reaction mixture (20 μL) contained 2 μL cDNA solution, 10 μL IQTMSYBRR Green Supermix (Bio-Rad, Hercules, CA, USA) and 6 μL each primer. The reactions were performed in triplicate, and the results were averaged. β-Actin was used as the reference gene. Relative gene expression was evaluated using the comparative cycle threshold method.

### DNA Extraction and 16S Gene Sequencing

DNA from different samples was extracted using the E.Z.N.A.®Stool DNA Kit (D4015, Omega, Inc., USA) according to the manufacturer's instructions. The reagent that was designed to uncover DNA from trace amounts of the sample has been shown to be effective for the preparation of DNA of most bacteria. Nuclear-free water was used for blank. The total DNA was eluted in 50 μL of elution buffer and stored at −80°C until PCR measurement.

The V3–V4 region of the prokaryotic (bacterial and archaeal) small-subunit (16S) rRNA gene was amplified with slightly modified versions of primers 338F (5′-ACTCCTACGGGAGGCAGCAG-3′) and 806R (5′-GGACTACHVGGGTWTCTAAT-3′). The 5′ ends of the primers were tagged with specific barcodes per sample and sequencing universal primers. PCR amplification was performed in a total volume of 25 μL reaction mixture containing 25 ng of template DNA, 12.5 μL PCR premix, 2.5 μL of each primer, and PCR-grade water to adjust the volume. The PCR conditions to amplify the prokaryotic16S fragments consisted of an initial denaturation at 98°C for 30 s; 35 cycles of denaturation at 98°C for 10 s, annealing at 54°C/52°C for 30 s, extension at 72°C for 45 s, and then final extension at 72°C for 10 min. The PCR products were confirmed with 2% agarose gel electrophoresis. Throughout the DNA extraction process, ultrapure water, instead of a sample solution, was used to exclude the possibility of false-positive PCR results as a negative control. The PCR products were purified by AMPure XT beads (Beckman Coulter Genomics, Danvers, MA, USA) and quantified by Qubit (Invitrogen, USA). The amplicon pools were prepared for sequencing, and the size and quantity of the amplicon library were assessed on Agilent 2100 Bioanalyzer (Agilent, USA) and with the Library Quantification Kit for Illumina (Kapa Biosciences, Woburn, MA, USA), respectively. PhiX control library (v3) (Illumina) was combined with the amplicon library (expected at 30%). The libraries were sequenced either on 300 PE MiSeq runs, and one library was sequenced with both protocols using the standard Illumina sequencing primers, eliminating the need for a third (or fourth) index read.

Samples were sequenced on an Illumina MiSeq platform according to the manufacturer's recommendations, provided by LC-Bio. Paired-end reads were assigned to samples based on their unique barcode and truncated by cutting off the barcode and primer sequence. Paired-end reads were merged using FLASH. Quality filtering on the raw tags was performed under specific filtering conditions to obtain the high-quality clean tags according to the fqtrim (V0.94). Chimeric sequences were filtered using Vsearch software (v2.3.4). Sequences with ≥ 97% similarity were assigned to the same operational taxonomic units (OTUs) by Vsearch software (v2.3.4). Representative sequences were chosen for each OTU, and taxonomic data were then assigned to each representative sequence using the Ribosomal Database Project (RDP) classifier. The differences in the dominant species in different groups were observed, and multiple sequence alignment was conducted using the MAFFT software (V7.310) to study the phylogenetic relationship of different OTUs. OTU abundance information was normalized using a standard sequence number corresponding to the sample with the least sequences. The data presented in the study are deposited in the NCBI BioProject PRJNA785429 repository, accession number from SAMN23561663 to SAMN23561687. Alpha diversity is applied in analyzing complexity of species diversity for a sample through 2 indices, including Chao1 and Shannon. All indices in our samples were calculated with QIIME (version 1.8.0). Beta diversity analysis was used to evaluate differences of samples in species complexity. Beta diversity was calculated by principle coordinate analysis (PCoA) and cluster analysis by QIIME software (version 1.8.0).

### Statistical Analysis

All experiments were conducted in triplicates. Data from each treatment were presented as the means ± standard deviation. One-way ANOVA using SPSS20 (IBM) assessed the mean differences between groups, followed by least significant difference (LSD) test, and different statistical significance was accepted at *p* < 0.05, *p* < 0.005, and *p* < 0.001.

## Results and Discussion

### Chemical Analysis of Instant Dark Tea

Instant dark tea contains 237.89 ± 3.13 mg·g^−1^ polysaccharide, 336.60 ± 5.67 mg·g^−1^ total polyphenol, 328.29 ± 0.51 mg·g^−1^ total flavonoids, 161.79 ± 6.08 mg·g^−1^ total catechins, 21.56 ± 0.57 mg·g^−1^ gallic acid, 5.86 ± 0.13 mg·g^−1^ free amino acid, 98.5 ± 3.29 mg·g^−1^ caffeine, and 4.22 ± 0.12 mg·g^−1^ theobromine. EGCG (46.89±1.81 mg·g^−1^), EGC (33.91±1.26 mg·g^−1^), ECG (21.31 ± 0.85 mg·g^−1^), EC (15.46±0.47 mg·g^−1^), GCG (14.65±0.55 mg·g^−1^), and GC (14.6±0.59 mg·g^−1^) were found to be the major catechins in IDT whereas C and CG were found in found at trace levels (<10.1 mg·g^−1^). Dark tea was rich in functional components, which contribute to its excellent antioxidant activity (30). The antioxidant activity of IDT using ORAC, DPPH, and ABTS was 94.3, 248.2, and 242.7 μM trolox·g^−1^, and this could lay the foundation on its lipid modulating effects.

### Effects of IDT on Body Weight and Metabolic Alterations Including Serum Lipid Level and Blood Glucose in HFD-Fed Rat

**Figure 1:**
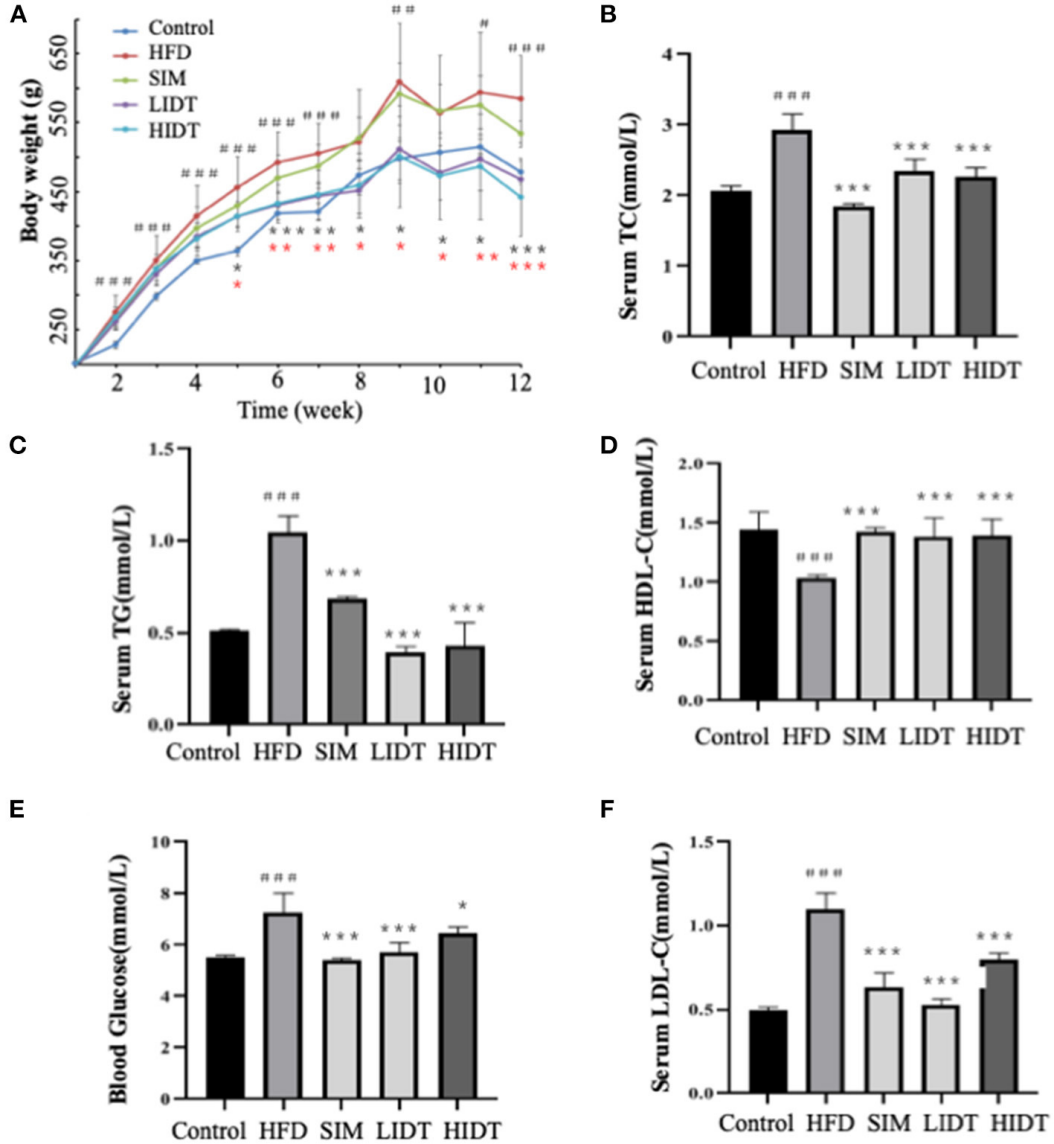
Effects of DTE on **(A)** body weight; **(B)** TG; **(C)** TC; **(D)** HDL-C; **(E)** LDL-C; **(F)** blood glucose. The different symbols represent significant differences between different groups. HFD, SIM, LIDT, HIDT vs. control, *p* < 0.05, #; *p* < 0.01,##; *p* < 0.005,###; SIM has no significant difference with HFD; LIDT vs. HFD, *p* < 0.05,*; *p* < 0.01,**; *p* < 0.005, ***; HIDT vs. HFD (* in red color), *p* < 0.05,*; *p* < 0.01,**; *p* < 0.005, ***.

Compared with control rat, HFD group gained more weight and developed hallmark features of disorder of lipid metabolism, including decreased HDL-C and elevated serum TG, TC, blood glucose, and LDL-C (*p* < 0.05) (Figure 1). As expected, supplementation with SIM and IDT significantly attenuated the HFD-induced effects. Attenuated weight gain was related to significantly reduced serum TG, TC, blood glucose, and LDL-C. An enhanced LDL-C level of SIM and IDT was also observed which contribute to lowing the weight gain. However, there is no obvious trend when different dosages with 0.27 and 0.54 g per kg bw were used, which might be because the low dose already achieved to positively modulate lipid metabolism. Taken together, these results demonstrated the ability of IDT to mitigate HFD-induced weight gain and hyperlipidaemia in rats.

### Effects of IDT on the Morphology of Liver Tissue in HFD-Fed Rat

**Figure 2:**
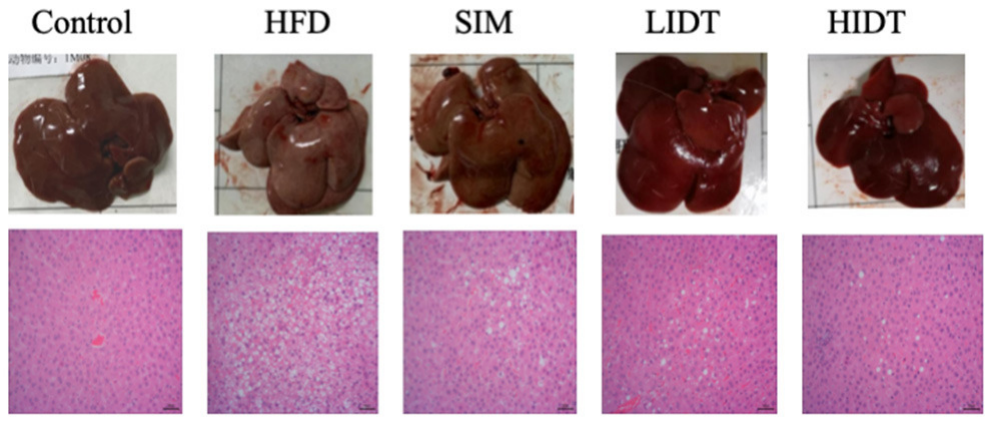
Effects of IDT on liver morphology in HFD-rat. The liver tissue was stained with H&E and observed at 200×.

The pictures and the microphotographs of H&E staining for liver tissues from the experimental mice are displayed in Figure 2. The liver in HFD-fed mice showed fat accumulation with yellow color in comparison with control, whereas IDT groups were similar with control. The liver tissue obtained from the control group exhibited normal hepatocytes (Figure 2). In contrast, the liver tissue from HFD-fed mice showed ballooned lipid laden hepatocytes, severe cellular degeneration, and the loss of cellular boundaries (Figure 2). The treatment with SIM presented inhibition effects against the hepatic injuries caused by HFD, which had significantly fewer vacuoles (Figure 2). Administration with IDT shows similar liver tissue structure with control, which shows a well-preserved cytoplasm, prominent nucleus, and legible nucleoli. These results documented that the intake of LIDT and HIDT in rats exhibited the improvement in HFD-induced fat deposition in liver.

### Effects of IDT on Hepatic Oxidative Stress and Damage in HFD-Fed Rat

**Figure 3:**
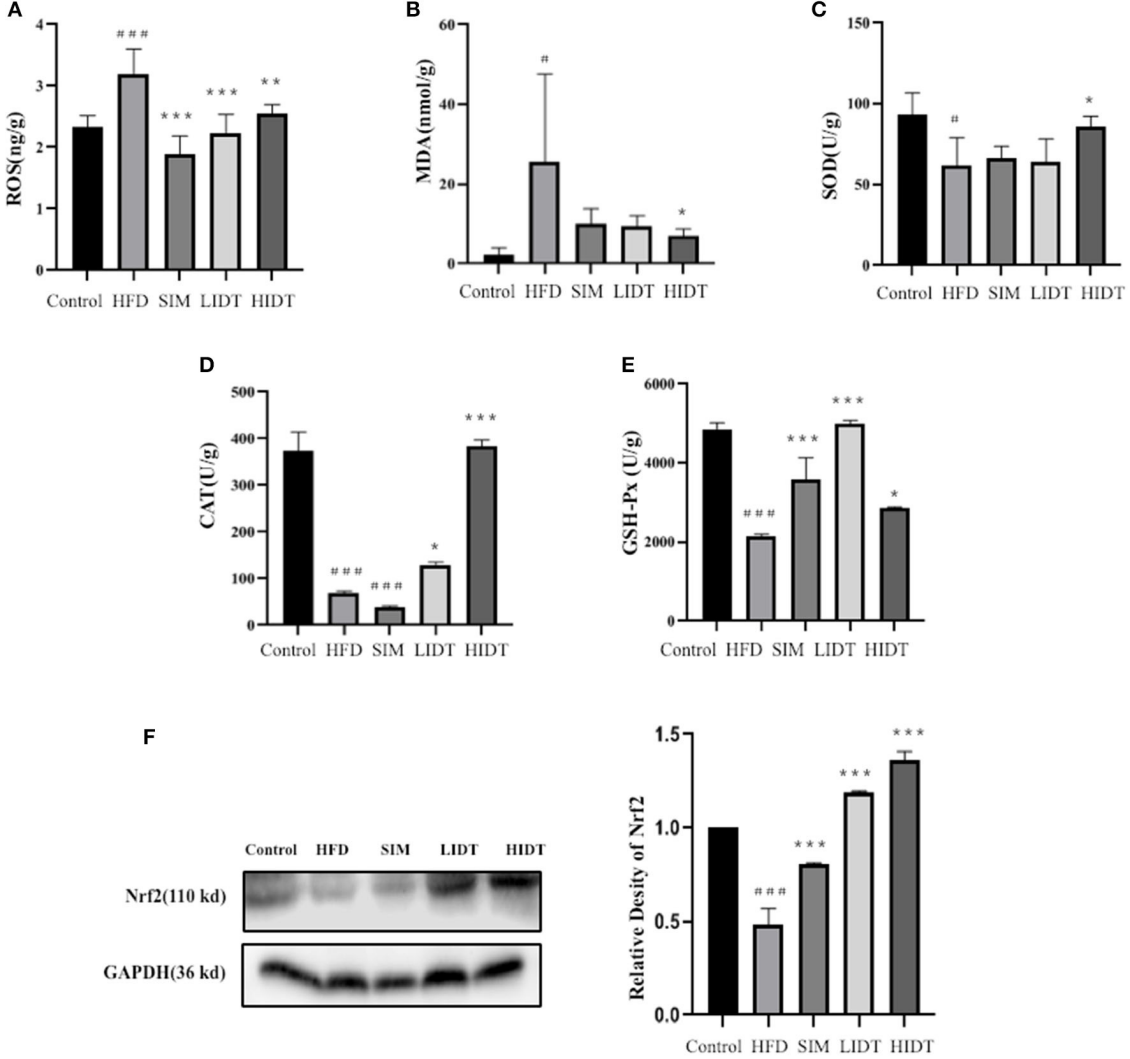
Effects of IDT on hepatic **(A)** ROS; **(B)** MDA; **(C)** SOD; **(D)** CAT; **(E)** GSH-Px; **(F)** Nrf2 protein expression. HFD, SIM, LIDT, HIDT vs. control, *p* < 0.05,#; *p* < 0.01,###; SIM, LIDT, HIDT vs. HFD, *p* < 0.05,*; *p* < 0.01,**; *p* < 0.005, ***.

As shown in Figure 3, the HFD group had a more significant severe liver oxidative stress level compared to control with a higher level of MDA and ROS and also a lower level of SOD, CAT, and GSH-Px (*p* < 0.05). The intervention with SIM and IDT prevented the hepatic oxidative stress indicators induced by HFD, and the IDT treatment was superior than that of SIM (Figure 3). HIDT significantly inhibited the ROS and enhanced SOD than that of HFD. There is no significant difference between control and HIDT with the level of MDA and CAT. The level of GSH-Px in LIDT was similar to the control. The protein expression of Nrf2 was inhibited in HFD and enhanced by our treatment than that of control. The decrease of MDA and ROS in combined effects of elevated levels of SOD, CAT, GSH-Px, and Nrf2 in IDT group which indicates that IDT was able to relieve hepatic oxidative stress.

### Effects of IDT on Hepatic Gene Relative Expression in HFD-Fed Rat

**Figure 4:**
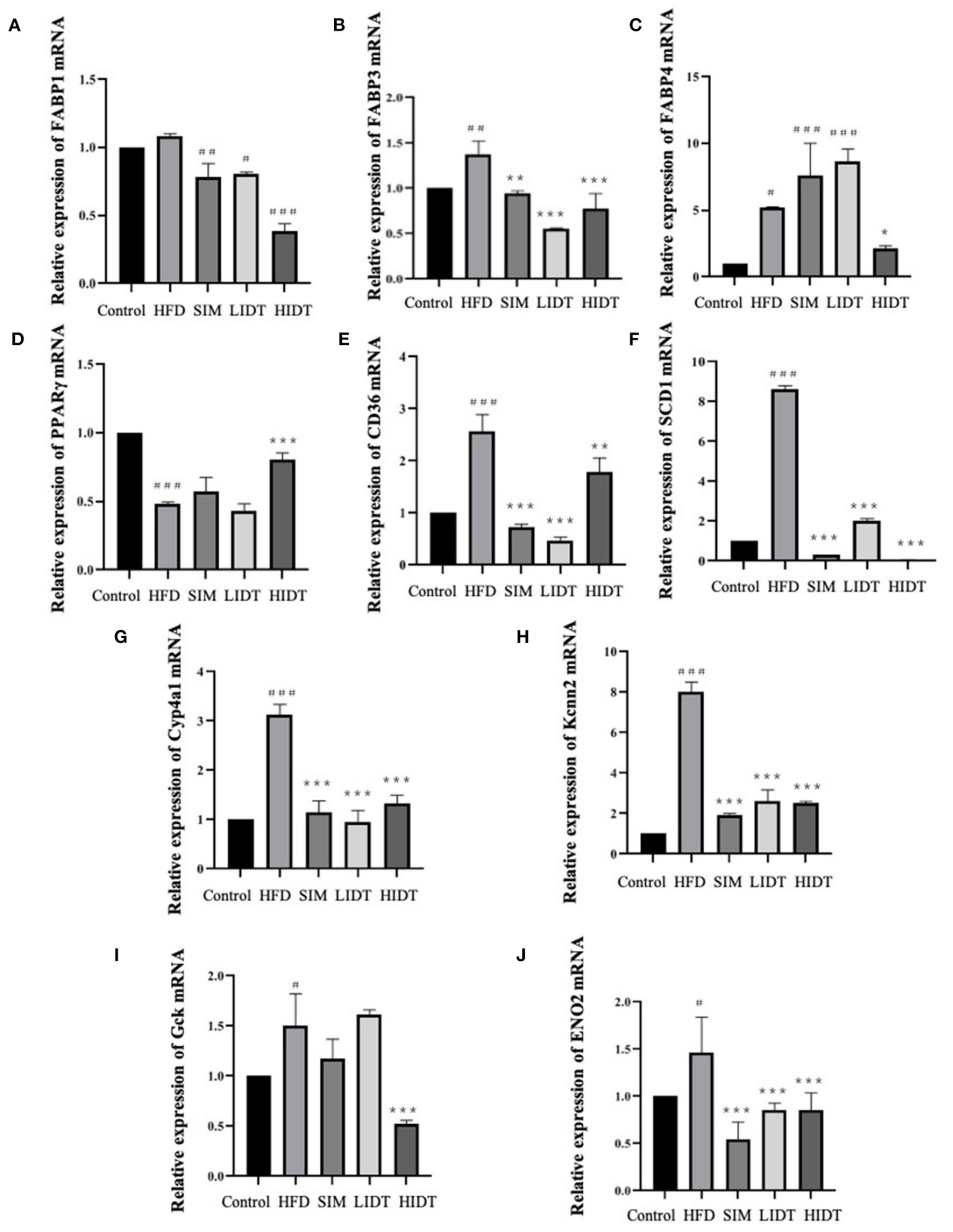
Effects of IDT on the hepatic relative expression of **(A)** *FABP1*; **(B)** *FABP3*; **(C)** *FABP4*; **(D)** *PPAR*γ; **(E)** *CD36*; **(F)** *SCD1*; **(G)** *Cyp4a1*; **(H)** *Kcnn2*; **(I)** *Gck*; **(J)** *ENO2*. HFD, SIM, LIDT, HIDT vs. control, *p* < 0.05,#; *p* < 0.01,##; *p* < 0.005,###; SIM, LIDT, HIDT vs. HFD, *p* < 0.05,*; *p* < 0.01,**; *p* < 0.005, ***.

The effect of IDT on the relative expression of lipid and glucose metabolism-related genes is shown in Figure 4. Gene expressions that increase the fatty acid uptake (*FABP1, FABP3, FABP4, SCD1, CD36, Cyp4a1, and Kcnn2*) and glycolysis (*Gck* and *ENO2*) were significantly (*p* < 0.05) enhanced after HFD diet than that of control, whereas significant decreases were observed in all treatments except that there is no effect on *FABP4* and *Gck* of SIM and LIDT group. *PPAR*γ is key for lipid oxidation and was significantly (*p* < 0.001) inhibited by HFD and restored by HIDT.

### Effects of IDT on Composition of Gut Microbiota

**Figure 5:**
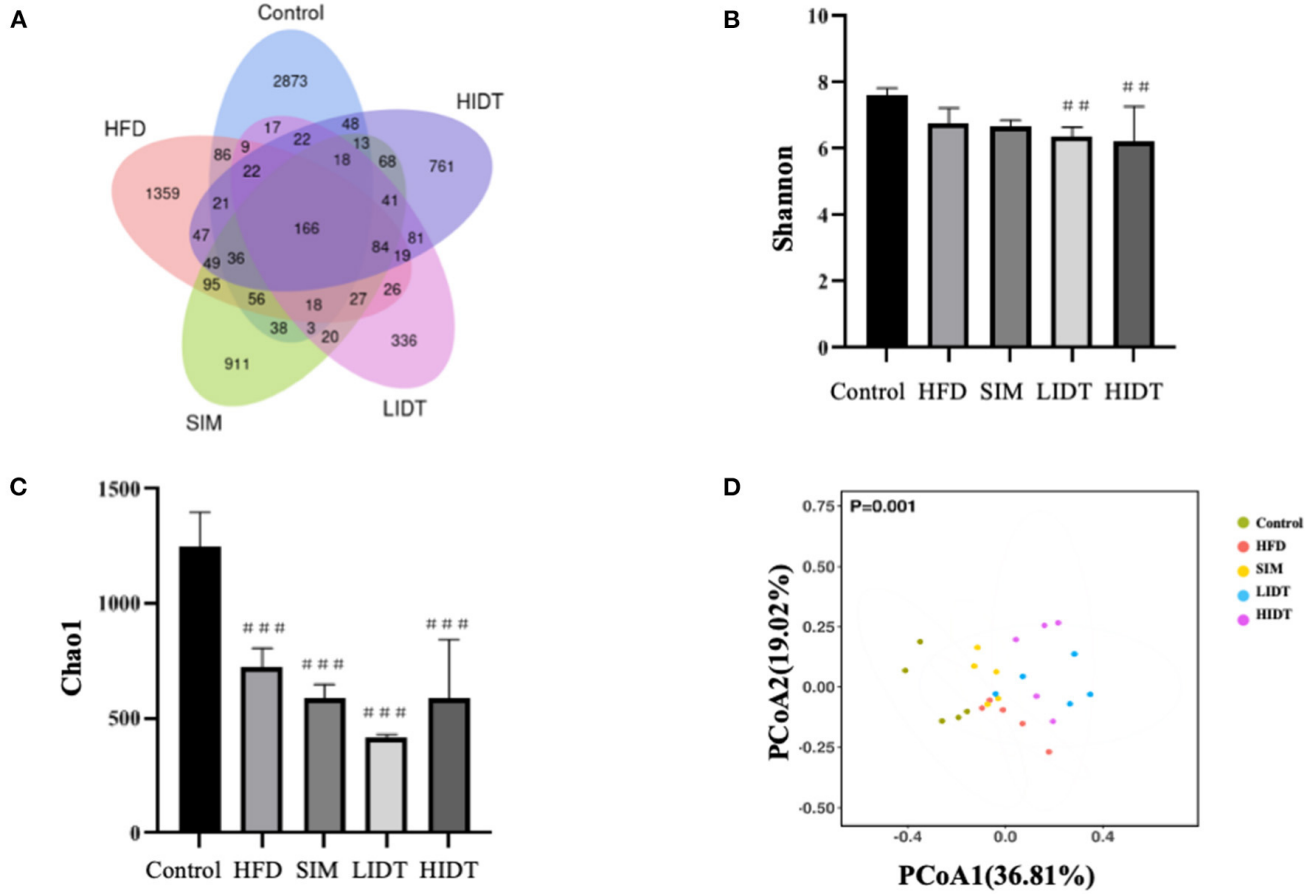
Effects of IDT on the composition of colon microbiota in HFD rats. **(A)** Venn diagram of three groups based on OUT; **(B)** A plot of the Shannon–Wiener diversity index; **(C)** a plot of the Chao 1 index; **(D)** the principal coordinates analysis (PCoA) score plot of colon microbiota in five groups. HFD, SIM, LIDT, HIDT vs. control, *p* < 0.01,##; *p* < 0.005,###.

To investigate how the gut microbiota is altered in rats, OTUs were compared among different groups (Figure 5). Although 166 OTUs were common among the groups, the control still had much more enriched sequences than other groups. The differences in OTUs were 87.92, 67.68, 59.18, and 70.32% for control, SIM, LIDT, and HIDT, respectively, in comparison with HFD, indicating that there was a different abundance of microorganisms among groups. Changes in alpha diversity of the gut microbiota community by administration of IDT are displayed in Figures 5B,C. The community evenness was presented with Shannon index whereas Chao 1 was used to evaluate the community microbiota richness. There is a decrease in Shannon index of all other four groups compared to control, but only significant for IDT groups (*p* < 0.05). The control also had the highest Chao 1 index than all other groups. These results showed that the microbiota community diversity and richness were reduced by the treatments when compared to control. PCoA could also display the microbial structure changes with clearly separated clusters (Figure 5D). Principal coordinate A1 (PCoA1) (percent variation explained 36.81%) could separate the control and other groups, whereas principal coordinate A2 (PCoA2) (percent variation explained 19.02%) could almost differentiate the HFD group from SIM and IDT groups.

To assess specific changes in the gut microflora, we compared the relative abundances of the predominant taxa identified in five groups at different levels. At the phylum level, a relative abundance of the top 10 species in each test is presented in Table 1. As expected, Bacteroidetes and Firmicutes were the majority relative abundance (>90%) of fecal inocula composition for the control, HFD, and SIM. After the supplementation with HFD, an increase in the relative abundance of Firmicutes and Proteobacteria (68.15 and 11.1%, *p* < 0.05) and a decrease in the relative abundance of Bacteroidetes and Cyanobacteria (26.17 and 0.1%, *p* < 0.05) in HFD in comparison with control were observed. While further treated with IDT, Firmicutes and Proteobacteria were significantly decreased. Interestingly, Verrucomicrobia was significantly increased by HIDT treatment with the relative abundance of 41.46%. HFD feeding resulted in a significant increase in the Firmicutes or Bacteroidetes (F/B, *p* < 0.005) ratio of 3.05. Fortunately, the increase in the F/B ratio induced by HFD was restored by SIM, LIDT, and HIDT treatment with the value of 1.17, 2.68, and 1.12, respectively.

**Table 1:** The intestinal microbiota at the phylum level.

**Phylum**	**Control**	**HFD**	**SIM**	**LIDT**	**HIDT**
Firmicutes	45.56 ± 11.95	68.15 ± 9.33 ↑#	49.47 ± 8.40 ↓*	55.02 ± 8.41	27.74 ± 5.73 ↓ (***,#)
Bacteroidetes	36.49 ± 4.17	26.17 ± 6.01 ↓#	35.66 ± 3.42 ↑*	9.38 ± 1.21 ↓***	25.01 ± 7.13 ↓#
Verrucomicrobia	0.04 ± 0.02	0.02 ± 0.03	0.36 ± 0.65	1.20 ± 1.33	41.46 ± 14.71 ↑###
Proteobacteria	1.58 ± 0.44	11.1 ± 1.22 ↑###	6.09 ± 0.77 ↓***	3.47 ± 0.99 ↓***	3.03 ± 0.48 ↓***
Actinobacteria	0.10 ± 0.07	0.24 ± 0.12	0.26 ± 0.25	1.33 ± 0.89	0.37 ± 0.17
Epsilonbacteraeota	0.37 ± 0.25	0.28 ± 0.40	0.90 ± 0.49	0.79 ± 1.70	0.18 ± 0.19
Cyanobacteria	4.03 ± 0.55	0.10 ± 0.11 ↓###	0.05 ± 0.02	0.07 ± 0.11	0.22 ± 0.32
Tenericutes	0.24 ± 0.26	0.19 ± 0.14	0.15 ± 0.10	0.23 ± 0.28	0.00 ± 0.01
Deferribacteres	0.01 ± 0.01	0.08 ± 0.13	0.05 ± 0.06	0.15 ± 0.21	0.09 ± 0.17
Elusimicrobia	0.62 ± 0.15	0.00 ± 0.01	0.00 ± 0.00	0.00 ± 0.00	0.00 ± 0.00
Others	1.81 ± 0.37	1.48 ± 0.80	1.17 ± 0.23	0.12 ± 0.22	0.20 ± 0.19
F/B	1.53 ± 0.00	3.62 ± 0.01 ↑###	1.14 ± 0.02 ↓ (***,##)	1.82 ± 0.11 ↓***	0.84 ± 0.09 ↓ (***,###)

The different symbols represent significant differences between groups. HFD, SIM, LIDT, HIDT vs. control, p < 0.05,#; p < 0.01,##; p < 0.005,###; SIM, LIDT, HIDT vs. HFD, p < 0.05,*; p < 0.005,***. ↑ stands for up-regulation; ↓ stands for down-regulation.

At the genus level, 30 genera identified with the highest relative abundance are presented in Table 2. HFD group resulted in significant increases in the genera of *Bacteroides, Clostridiales, Bilophila*, and *Ruminococcus_1* and was accompanied by significant decreases in the genera of *Muribaculaceae, Rikenellaceae_RC9_gut_group, Prevotellaceae_UCG001, Bacteroidetes*, and *Ruminococcaceae_UCG-014*. Relative to the control group, the percentage of *Clostridiales, Ruminococcaceae_UCG-005*, and *Ruminococcus_1* significantly reduced whereas *Rikenellaceae_RC9_gut_group* significantly increased by the treatment of SIM, LIDT, and HIDT. Interestingly, the supplement with HIDT had a significant increase in *Akkermansia*, which also significantly decreased the relative abundance of *Lachnospiraceae* and *Lachnospiraceae_UCG-010* (*p* < 0.005) which increased by HFD.

**Table 2:** The intestinal microbiota composition at genus level.

**Genus**	**Control**	**HFD**	**SIM**	**LIDT**	**HIDT**
*Bacteroides*	2.44 ± 0.59	9.66 ± 1.46 ↑##	22.60 ± 5.69 ↑***	7.67 ± 1.35 ↑#	12.12 ± 0.28 ↑###
*Akkermansia*	0.02 ± 0.01	0.02 ± 0.02	0.36 ± 0.65	0.43 ± 0.11	49.71 ± 5.02 ↑(***,###)
*Ruminococcaceae_UCG-005*	6.86 ± 4.60	19.34 ± 4.45 ↑###	6.09 ± 2.40 ↓***	3.60 ± 0.84 ↓***	2.15 ± 0.79 ↓***
*Clostridiales*	0.65 ± 0.68	9.25 ± 1.46 ↑###	4.52 ± 3.48 ↓*	2.64 ± 0.77 ↓***	8.72 ± 2.52
*Muribaculaceae*	12.82 ± 6.30	4.28 ± 1.46 ↓##	3.60 ± 0.95 ↓###	1.72 ± 2.78 ↓###	2.79 ± 1.31 ↓###
*Rikenellaceae_RC9_gut_group*	4.76 ± 0.59	0.36 ± 0.10 ↓###	6.54 ± 0.48 ↑***	0.68 ± 0.48 ↓###	3.42 ± 1.40 ↑***
*Ruminiclostridium_9*	0.84 ± 0.33	3.69 ± 0.69 ↑#	2.61 ± 2.21	2.88 ± 1.25	1.34 ± 0.42 ↓*
*Firmicutes*	4.44 ± 1.10	4.91 ± 1.32	2.37 ± 0.73 ↓(#,*)	0.69 ± 0.28 ↓(###,***)	1.50 ± 1.67 ↓(#,***)
*Lachnospiraceae*	1.44 ± 0.45	2.71 ± 1.26 ↑#	1.99 ± 0.24	2.67 ± 0.49 ↑#	0.65 ± 0.21 ↓***
*Parabacteroides*	0.55 ± 0.20	0.73 ± 0.07	1.45 ± 0.68	1.50 ± 0.66	1.46 ± 0.68
*Alloprevotella*	4.38 ± 1.15	3.46 ± 0.65	4.42 ± 1.33	1.06 ± 1.55 ↓##	2.37 ± 1.16
*Bilophila*	0.41 ± 0.42	3.37 ± 1.51 ↑##	3.89 ± 1.16 ↑###	2.30 ± 0.69	2.33 ± 1.32
*Intestinimonas*	1.65 ± 0.83	1.90 ± 0.40	1.37 ± 0.31	2.20 ± 0.35	1.91 ± 0.43
*Blautia*	0.06 ± 0.01	1.08 ± 0.41	2.24 ± 0.86	2.70 ± 2.73 ↑#	0.34 ± 0.20
*Ruminococcaceae*	0.46 ± 0.20	0.72 ± 0.23	0.76 ± 0.32	1.26 ± 0.23 ↑#	1.38 ± 0.72 ↑#
*Lachnospiraceae_NK4A136_group*	3.43 ± 2.21	3.14 ± 0.38	0.74 ± 0.63 ↓(*,#)	0.33 ± 0.43 ↓(*,##)	0.59 ± 0.59 ↓(*,#)
*Roseburia*	0.88 ± 0.56	1.53 ± 0.45	1.26 ± 0.66	1.22 ± 0.38	0.68 ± 0.33
*Romboutsia*	0.22 ± 0.15	0.79 ± 0.06	1.08 ± 0.58	2.67 ± 1.33 ↑(**,###)	0.96 ± 0.49
*Ruminococcus_1*	1.86 ± 0.84	3.91 ± 2.74 ↑	0.63 ± 0.54 ↓*	0.01 ± 0.01 ↓***	0.01 ± 1.01 ↓***
*Prevotellaceae_UCG-001*	5.11 ± 1.30	0.38 ± 0.24 ↓###	0.75 ± 0.14 ↓###	0.02 ± 0.02 ↓###	0.31 ± 0.09 ↓###
*Phascolarctobacterium*	1.20 ± 0.71	1.40 ± 0.62	2.09 ± 0.46	0.74 ± 0.56	1.22 ± 0.46
*Desulfovibrionaceae*	1.33 ± 1.14	0.30 ± 0.20	0.46 ± 0.50	0.56 ± 0.40	0.75 ± 0.23
*Bacteroidetes*	5.75 ± 4.28	0.40 ± 0.07 ↓##	0.59 ± 0.11 ↓##	0.20 ± 0.06 ↓##	0.44 ± 0.01 ↓##
*Subdoligranulum*	0.07 ± 0.07	0.33 ± 0.07	0.42 ± 0.19	2.95 ± 1.94 ↑###	0.43 ± 0.47
*Erysipelatoclostridium*	0.22 ± 0.16	0.34 ± 0.16	0.14 ± 0.12	2.08 ± 2.68 ↑#	1.07 ± 0.64
*Ruminococcaceae_UCG-014*	3.28 ± 0.80	1.42 ± 0.30 ↓###	0.91 ± 0.36 ↓###	0.20 ± 0.09 ↓###	0.05 ± 0.05 ↓###
*Ruminococcaceae_NK4A214_group*	0.61 ± 0.01	1.04 ± 0.13	0.56 ± 0.24	1.27 ± 0.59	1.91 ± 1.6
*Negativibacillus*	0.18 ± 0.07	0.43 ± 0.16	0.25 ± 0.12	1.64 ± 1.38 ↑*	0.69 ± 0.40
*Lachnospiraceae_UCG-010*	0.16 ± 0.15	1.86 ± 0.52 ↑###	1.54 ± 0.62 ↑###	1.54 ± 0.51 ↑###	0.33 ± 0.33 ↓***
*Clostridium_sensu_stricto_1*	0.01 ± 0.02	0.34 ± 0.11	0.51 ± 0.18	3.90 ± 4.91	0.89 ± 0.96
Others	27.92 ± 2.09	19.28 ± 5.23	17.38 ± 2.45	21.66 ± 6.16	15.58 ± 10.12

The different symbols represent significant differences between groups. HFD, SIM, LIDT, HIDT vs. control, p < 0.05,#; p < 0.01,##; p < 0.005,###; SIM, LIDT, HIDT vs. HFD, p < 0.05,*; p < 0.01,**; p < 0.005,***. ↑ stands for up-regulation; ↓ stands for down-regulation.

**Figure 6:**
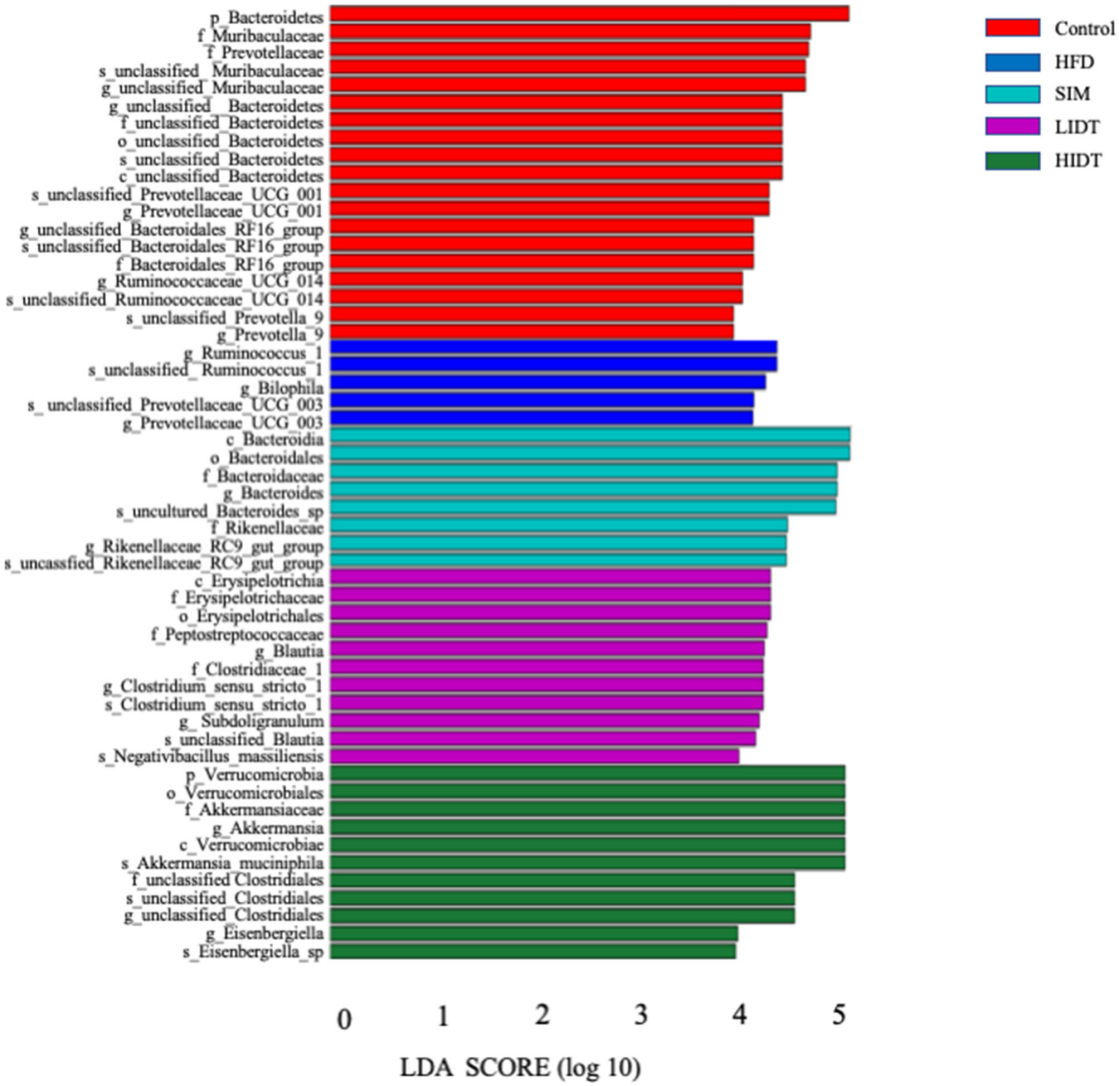
Linear discriminative analysis (LDA) effect size (LEfSe) analyses of statistically significant taxonomies between groups. The vertical coordinate is the taxa with significant differences between groups, and the horizontal coordinate is the bar graph to show the LDA difference analysis of each species group. The scores of LDA) value which is >4 are sorted according to the scores, so as to describe their differences in different groups of samples. The longer the length is, the more significant the difference is.

Linear discriminant analysis (LDA) effect size (LEfSe) algorithm, as a metagenomic biomarker discovery approach, was performed to further identify the specific bacterial taxa at species level that differentially in response to the different diet interventions. Taxa with LDA score threshold >4 are shown in Figure 6. LEfSe detected 19, 8, 11,11, and 5 bacterial branches in the fecal flora of control, SIM, LIDT, HIDT, and HFD, respectively, and the differences were statistically significant. The control was dominated by genus *Bacteroidetes, Muribaculaceae*, and *Prevotellaceae* whereas the most abundant bacterial groups in the HFD group belong to the genus *Ruminococcus, Prevotellaceae*, and *Bilophila*. Different dosages of IDT had distinct marker bacteria. The LIDT group was characterized by the genus *Clostridium* and *Blautia* and also the family Peptostreptococcaceae and Erysipelotrichaceae, whereas order Verrucomicrobiales, genus *Akkermansia, Eisenbergiella*, and *Clostridiales* were the most dominant bacteria in HIDT group.

**Figure 7:**
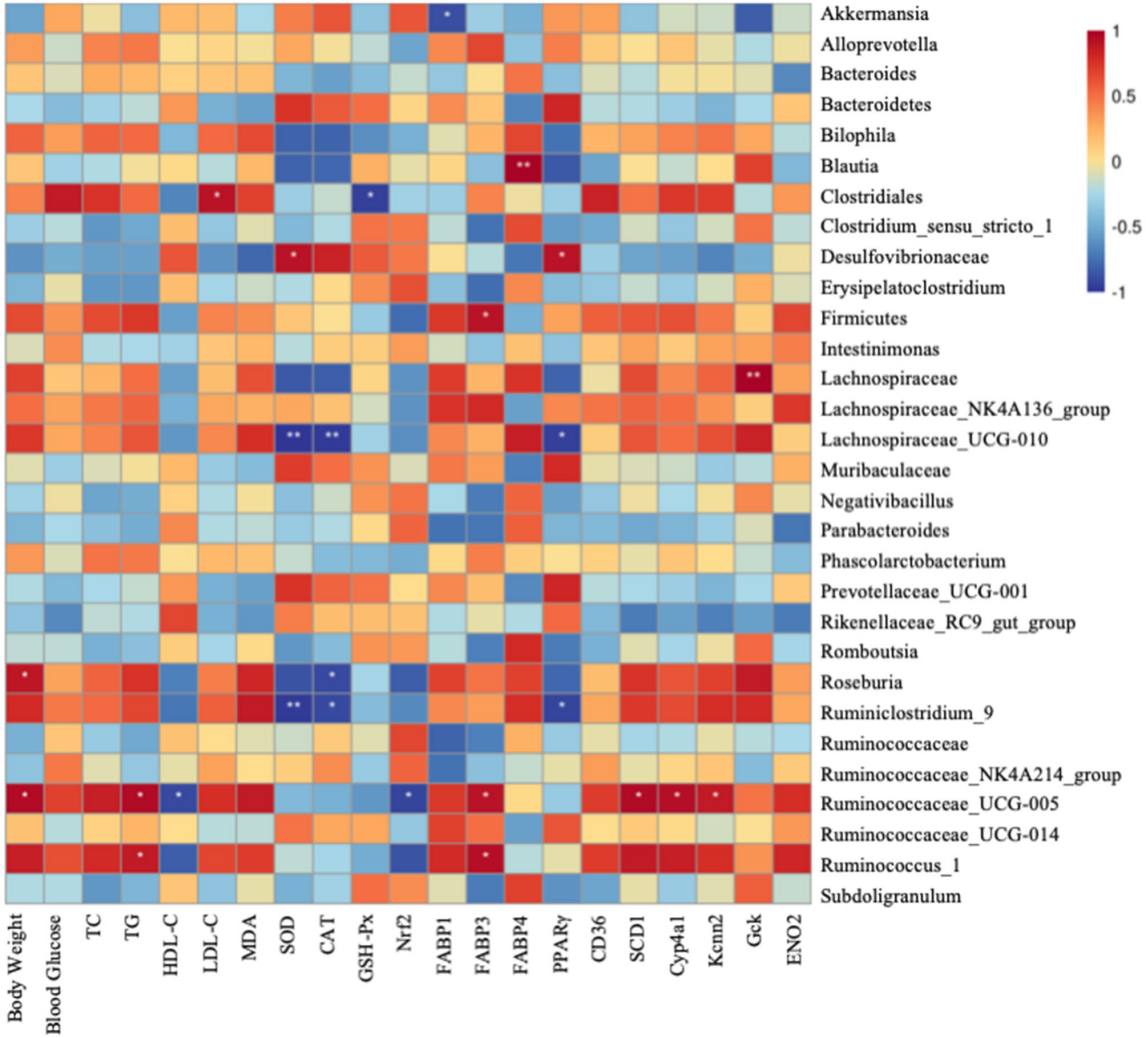
Spearman's correlation between gut microbiota at genus level and biochemical parameters and lipid metabolism genes. Colors of squares represent *R*-value of Pearson's correlation. *p* < 0.05* and *p* < 0.01** indicate the significant level in the correlation, respectively.

Finally, a heatmap analysis of genera correlated with biochemical parameters including body weight, serum lipid level, oxidative level, and metabolism gene expression is shown in Figure 7. *Lachnospiraceae_UCG-010* and *Ruminiclostridium_9* were significantly correlated with SOD and CAT. *Clostridiales* highly correlated with blood glucose, LDL-C, and MDA, whereas positively correlated with GSH-Px and HDL-C. *Ruminococaceae-UCG-005* significantly negatively correlated with HDL-C and Nrf2. *Lachnospiraceae-UCG-010* and *Ruminococaceae-UCG-005* had significant correlation with MDA and TG. All these indicate that gut microbiotas might be associated with oxidative stress and fatty liver (Figure 7). *Akkermansia* was significantly negatively correlated with *FABP1*. *Firmicutes, Ruminociccaceae_UGG-005*, and *Ruminocuccus_1* were significantly positively related to *FABP3*. *Blautia* was significantly positively correlated with *FABP4*. *Lachnospiraceae_UCG-010* and *Ruminiclostridium_9* were significantly negatively correlated with *PPAR*γ whereas it is significantly positively correlated with *Desulfovibrionaceae*. *Ruminociccaceae_UGG-005* was also significantly correlated with *SCD1, Cyp4a1*, and *Kcnn2*. *Gck* was significantly positively correlated with *Lachnospiraceae*. All these findings suggest that IDT may alleviate the hepatic oxidation and gene expression by regulating the diversities of intestinal microbiota.

## Discussion

Our present results showed that continuously 12-week ingestion of HFD feeds led to obesity and hyperlipidaemia in rat (Figure 1). Hyperlipidaemia characterizing with abnormal lipid metabolism is the dominant causing of chronic diseases, including obesity, diabetes, and cardiovascular and cerebrovascular diseases (2). Until now, it can be treated by a combination of medicine, foods, or physical exercise (31). Dark tea as a particular drink significantly affects the lipid metabolism, which shows antiobesity effects. IDT is a new tea product that conforms to the fast-paced life in modern society as easy to drink, exerting higher antioxidant activity compared to normal dark tea extract, which also approved to contain higher content of bioactive compounds such as polysaccharides, polyphenols, and caffeine (32). Tea polysaccharides are considered as the main bioactive ingredients in tea, modulating the metabolic disease including antioxidant and antiinflammatory effects, inhibition of digestive enzymes, prevention of macronutrient absorption, expression of gene and protein, and also interaction with gut microbiota (33). Polyphenols are the primary functional ingredient in tea and play a role in scavenging oxygen free radicals and changing gut flora (34). The content of polyphenol and catechins in our study was higher than that of reported content in various dark tea water extract with polyphenol content that ranged from 11.61 to 18.22% and catechin content from 4.81 to 6.83% (35). EGCG, as the most abundant catechin presented in tea, has been widely accepted for its lipid-lowering effects in animal model and clinical research (36, 37). The caffeine content was in consistent with the reported content in instant dark tea with around 8% (38). Polysaccharides, polyphenols, and caffeine have inhibitory effects on body weight increase and fat accumulation, of which polysaccharides and polyphenols were presenting synergistic effects (39). As the polyphenols have poor bioavailability, polysaccharides could act as a nature carrier (40). A study recent showed that the polyphenols could be the major contributor to the functionality of polysaccharides (41). Gallic acid (GA) is a primary polyphenol in dark tea, and it is known to have antihyperlipidemic effect (9). GA significantly suppressed the weight gain and serum lipid parameters including TG and TC through inhibiting the pancreatic lipase activity (42). Thus, IDT has higher functional ingredients, following higher antioxidant activity, which is likely responsible for its preventing effects on obesity and hyperlipidaemia.

**Figure 8:**
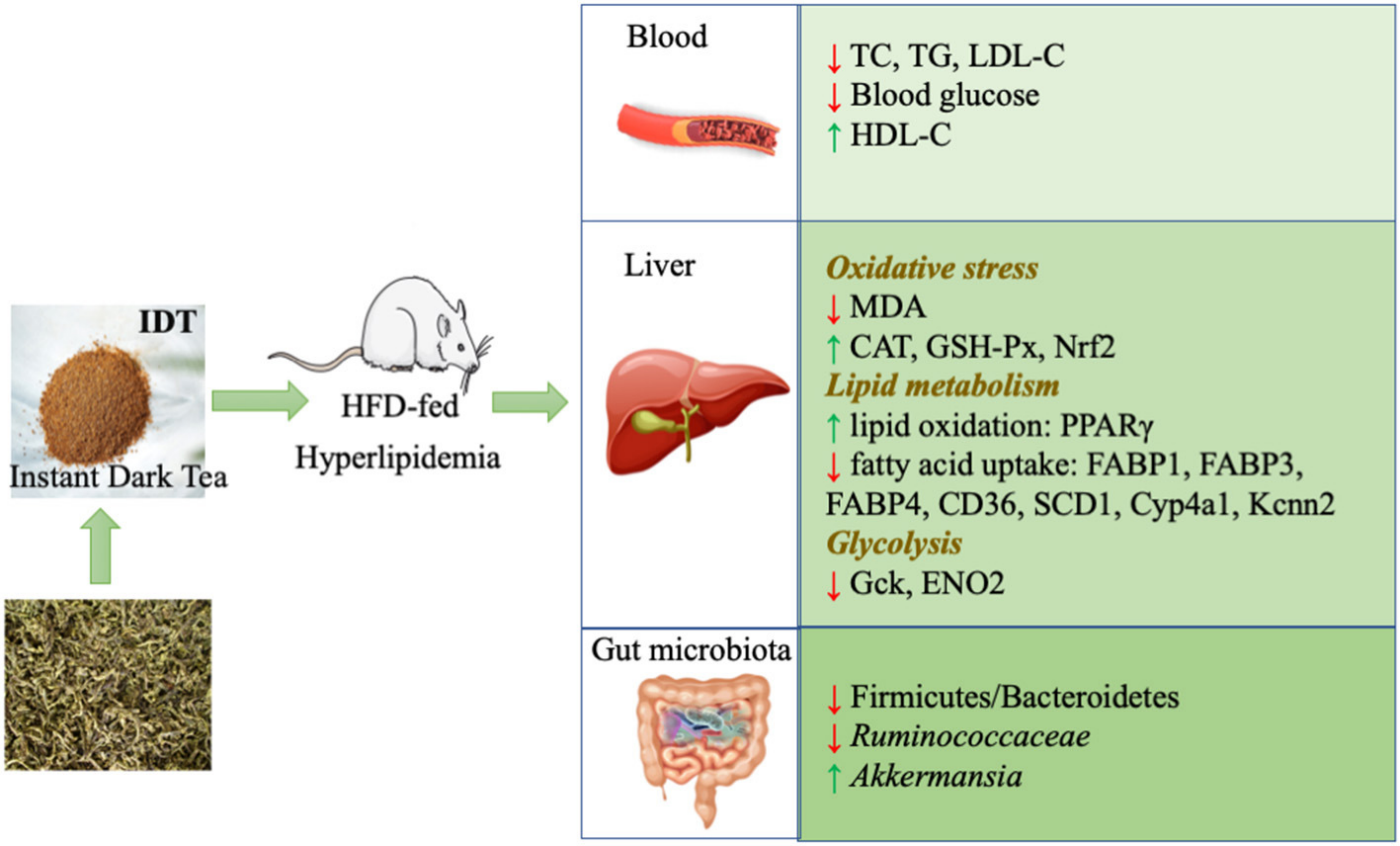
Attenuation mechanism of IDT on hyperlipidaemia.

Our study found that consumption of IDT merged in HFD feeds in rat could antagonize the abnormal lipid level through regulating the oxidative stress, metabolism genes, and gut microbiota, as displayed in Figure 8. Many studies have reported that HFD is able to induce abnormal lipid accumulation, leading to obesity and hyperlipidaemia. In our hand, IDT intake was significantly induced the increases in the serum TC, TG, and LDL-C and the decrease in the serum HDL-C levels in comparison with the untreated normal rat. Histological observations showed that an increased hepatic vacuolisation and lipid droplets in HFD and IDT treatment were similar to the control. Oxidative stress is a state of imbalance between the oxidative and antioxidative systems of the cells and tissues, which leads to elevated level of ROS that cause damage to lipids, proteins, and DNA (43). MDA is a typical lipid peroxidation marker (44). SOD, CAT, and GSH-Px play an important role on defending against oxidative stress. Hepatic oxidative stress is crucial in the pathogenesis of hyperlipidaemia. In our study, consumption of IDT decreased the hepatic MDA and enhanced the antioxidant enzyme (SOD, CAT, and GSH-Px) activities against the damage from HFD, which is similar to the reported studies treated by dark tea (20, 45). The observed high level of antioxidant enzyme is likely linked to the Nrf2 activation. EGCG prevents HFD damage through modulation of key regulating detoxifying enzymes *via* regulation of Nrf2 function (46). More importantly, this effect could partly *via* the regulation of specific significantly correlated gut microbiota including *Clostridiales, Ruminococaceae-UCG-005, Lachnospiraceae_UCG-010, Ruminiclostridium_9, and Ruminocuccus_1*. *Clostridiales, Ruminiclostridium_9*, and *Ruminocuccus_1* were reported positively correlated with high-fat intake (47, 48). *Ruminococcaceae_UCG-005* positively correlated with MDA level after HFD treatment, which also showed in our study (49). The *Lachnospiraceae_UCG-010* was associated with an increase in blood glucose level (50), which was significantly decreased by HIDT compared to HFD.

Hepatic lipid metabolism plays a key role in controlling the whole-body lipid homeostasis. The strong modulation of IDT on host liver genes including lipogenesis (*FABP1, FABP3, FABP4, SCD1, CD36, Cyp4a1, and Kcnn2*), lipid oxidation (*PPAR*γ), and glycolysis (*Gck, ENO2*) involved in lipid and glucose metabolism was also observed. To our knowledge, this is the first report to reveal the gene target of IDT on inhibiting lipid synthesis and accelerating lipid β-oxidation and glycolysis in the liver. Fatty acid-binding proteins (*FABPs*) are versatile proteins that can modulate lipid metabolism (8). *FABP1* is a liver-specific fatty acid-binding protein that plays important roles in intracellular lipid metabolism in the liver, and the knockdown of *FABP1* in liver decreased the liver weight and hepatic TG (51). *FABP3* seems to be lipid metabolism-related biomarker in Alzheimer's disease (52). Markedly, increase in *FABP3* mRNA expression in rats' livers and adipose tissue was reported with high-cholesterol diet (53). *FABP3* has been shown to be markedly upregulated in high-fat-induced mice and zebrafish (54, 55). A citrus bioflavonoid named hesperidin ameliorated liver steatosis in high-cholesterol diet rats through downregulation of *FABP3* (56). Elevated *FABP4* levels are associated with obesity and metabolic disease (57). All measured *FABP* families were inhibited by the treatment of HIDT, compared to control. Consumption of tea polyphenol EGCG inhibited FABP to reduce fat deposit in mice and rats (58). *PPAR*γ plays a significant role in protecting the liver from inflammation, oxidation, fibrosis, fatty liver, and tumors, and the activation of *PPAR*γ could stimulate fatty acid oxidation in the liver (59). Administration of green tea polyphenols decreased mRNA and protein expressions of *PPAR*γ and adiponectin in high-fat-fed rats (60). *PPAR*γ was enhanced in HFD-induced mice by the treatment of polysaccharides from flaxseed (61). In this study, HIDT is a promising natural product for *PPAR*γ-activating. *SCD1* is a rate-limiting enzyme catalyzing to increase the formation of TG, and this significantly decreased by the polyphenol and polysaccharide, whereas *PPAR*γ was reported to be inhibited (62, 63). Meanwhile, *CD36* and *CYP4A1* genes were also a common downstream target of PPARγ, which was significantly decreased by natural antioxidant (64). *Kcnn2* was enhanced in high-fat diet rats (65). In addition, our result showed that IDT also modulated the glucose production. This effect was associated with the glycolysis genes including *GCK* and *ENO2*. Taken together, these results indicate that the ability of IDT to alleviate hyperlipemia may decrease lipid lipogenesis and enhance fat catabolism and oxidation, and this effect might associate with the modulation of intestinal flora.

Diet nutrition is known to be essential on the composition of intestinal microbiota, which have strong influence on human health (66). Proteobacteria is a marker for an unstable microbial community (dysbiosis) and a potential diagnostic criterion for disease (67), which was significantly decreased by out treatment. As expected, the HFD-induced increase in F/B ratio was visibly counteracted by IDT treatment (Table 2), which is similar to the results obtained from Fu instant tea and Fu brick tea (20, 32). What is more, IDT dramatically modified the gut microbial species at the genus level with the decrease of *Clostridiales, Ruminococcaceae_UCG-005, Ruminococcus_1, Ruminococcaceae_UCG-014, Lachnospiraceae*, and *Lachnospiraceae_UCG-*010 and a significant increase in beneficial bacteria *Akkermansia* and *Rikenellaceae_RC9_gut_group*. *Ruminococcaceae_UCG-005*, as a harmful biomarker in gut microbiota (20), was significantly negatively correlated with HDL-C and positively correlated with body weight, TG, *FABP3, SCD1, Cyp4a1*, and *Kcnn2*, which could serve as target to modulate the lipid metabolism. *Lachnospiraceae* contributes to the onset of metabolic dysfunction (68). The relative abundance of *Rikenellaceae_RC9_gut_group* was enhanced by SIM and HIDT, which was shown to be decreased in rats with hypertriglyceridemia-related acute necrotising pancreatitis (69). It was reported that *Akkermansia* metabolites affects various transcription factors and genes such as *PPAR*γ involved in growth and cellular lipid metabolism (70). *Akkermansia* was negatively correlated with *FABP1*. The proliferation of *Akkermansia* by IDT supplementation may partially contribute to the regulation of lipid metabolism involved genes in liver and finally prevent liver tissue fat deposition in HFD rats. Thus, the level of lipid metabolic genes and composition of microbiota in IDT-treated rats were greatly different from the HFD group, which indicates that supplementation of IDT was potentially an effective way for regulating hyperlipidaemia-associated disorder.

Thus, IDT contains various phytochemicals, which could be utilized by the gut microbiota to produce the metabolic substrates and various bioactive metabolites. These components were absorbed through the portal vein into the liver, to regulate hepatic oxidative stress and lipid metabolism.

## Conclusion

The results of this study demonstrated that IDT, as the convenience consuming form, obviously improved the obesity and lipid metabolism disorder in HFD-induced rats *via* mitigating oxidative stress (Nrf2 and antioxidant enzymes), lipid metabolism (*PPAR*γ-*CD36*-*SCD1*), glucose metabolism (*Gck* and *ENO2*), and gut microbiota (enhancement of beneficial gut bacteria *Akkermansia*). In addition, seven important genera (*Akkermansia, Clostridiales, Lachnospiraceae, Lachnospiraceae_UCG-010, Ruminiclostridium_9, Ruminococaceae-UCG-005*, and *Ruminocuccus_1*) of intestinal flora was significantly correlated with oxidative stress and lipid metabolism and could be the potential biomarkers. In the future, understanding of the deeper mechanism of intestinal microbiota mediating the lipid disorder of IDT is an attractive challenge and yet requires further investigation on germ-free mice or in antibiotic-treated mice. Moreover, it would be of interest to determine the bioconversion of IDT by the superorganism of hosts and microbiota and subsequently ensure whether these metabolites contribute to its functionality.

## Data Availability Statement

The original contributions presented in the study are publicly available. This data can be found here: https://www.ncbi.nlm.nih.gov/sra/PRJNA785429.

## Ethics Statement

This study was reviewed and approved by Hunan Research Center for Safety Evaluation of Drugs, SYXK2015-0016.

## Author Contributions

ZH wrote the original draft. ZZ, HZ, and CL performed the experiments and collected the samples. YW, CZ, YY, and JY contributed in methodology and data analysis. SQ and HW participated in supervision. SQ, ZL, and MS contributed in project administration and conceptualization, writing, reviewing, and editing. All authors listed have made a substantial, direct, and intellectual contribution to the work and approved it for publication.

## Funding

This work was supported by Hunan Province Innovative Postdoctoral Project (2021RC2080).

## Conflict of Interest

YW, YY, and HW were employed by Hunan Tea Group Co. Ltd. The remaining authors declare that the research was conducted in the absence of any commercial or financial relationships that could be construed as a potential conflict of interest.

## Publisher's Note

All claims expressed in this article are solely those of the authors and do not necessarily represent those of their affiliated organizations, or those of the publisher, the editors and the reviewers. Any product that may be evaluated in this article, or claim that may be made by its manufacturer, is not guaranteed or endorsed by the publisher.
