# Supplementary material for: Instant Dark Tea Alleviates Hyperlipidaemia in High-Fat Diet-Fed Rat: From Molecular Evidence to Redox Balance and Beyond
Source: Front Nutr. 2022 Feb 3;9:819980. doi: 10.3389/fnut.2022.819980 (PMC8875000; doi:10.3389/fnut.2022.819980)
Supplement: Supplementary file 1 [file Table_1.DOCX]

| Gene | Forward primer (5‘-3‘) | Reverse primer (3‘-5‘) |
| --- | --- | --- |
|  |  |  |
| *FABP1* | TCTCCGGCAAGTACCAAGTG | GCCTTGACCTTTTCCCCAGT |
| *FABP3* | CAAGCCGACCACAATCAT | AGTTTGCCTCCGTCCAGT |
| *FABP4* | AGAAGTGGGAGTTGGCTTCG | TCATGACACATTCCACCACCA |
| *PPARγ* | GGCTGCAGCGCTAAATTCAT | GTCAGCTCTTGTGAACGGGA |
| *CD36* | CTCCCCACTCCAGAACCCAGAC | CCAGCACACCATACGACGTACAG |
| *SCD1* | TGTCAAAGAGAAGGGCGGAAAGC | CAGGATGAAGCACATGAGCAGGAG |
| *Cyp4a1* | CTCCGTGCTTGGTCTGCTTCTG | GAGGTGATGGGAACTGCTGGAAAG |
| *Kcnn2* | TGGATAATTGCCGCATGGACTGTC | TTGCTCCAAGGAAGTTGCTAGTGAC |
| *Gck* | GAGCAGAAGGGAACAACATCGTAGG | TCGTTCACCATTGCCACCACATC |
| *ENO2* | GGGACAAACAGCGTTACTT | TCCAACTCCAGCATCAGG |

TABLE S1 Primer sequences for PCR.
